# Digitising wound care: a cost-consequence analysis of the Wound Care Command Centre™ in Australia

**DOI:** 10.1186/s12913-025-12969-2

**Published:** 2025-07-01

**Authors:** Michelle Barakat-Johnson, Meg Newton, Crispin Cayley, Michelle Lai, Laura Dixie, Nathaniel Alexander, Marc Pelusi, Jimmy Chan, Anna Cohen

**Affiliations:** 1 Nursing and Midwifery Executive Services, Sydney Local Health District, Level 11 King George V Building, Nursing Executive, Missenden Road Camperdown, Sydney, NSW Australia; 2 Susan Wakil School of Nursing and Midwifery, Faculty of Medicine and Health, University of Sydney, Camperdown, Sydney, NSW Australia; 3 School of Health and Human Sciences, University of Huddersfield, Huddersfield, UK; 4 Taylor Fry, Sydney, Australia; 5 South Western Sydney Local Health District, Liverpool Hospital Eastern Campus, Liverpool, Sydney, NSW Australia; 6 Single Digital Patient Record Implementation Authority, Chatswood, Sydney, NSW Australia; 7 RPA Virtual Hospital, Sydney Local Health District, Camperdown, Sydney, NSW Australia

**Keywords:** Cost-consequence, Chronic wounds, Digital wounds, Wound care, Wound care technology

## Abstract

**Background:**

Chronic wounds pose considerable financial challenges for healthcare systems globally, with most cases requiring hospital care and extended lengths of stay, particularly due to delayed access to treatment. To address this, Sydney Local Health District (LHD) in Australia launched the Wound Care Command Centre™ in 2023, utilising a digital application for timely access to wound care and to reduce the burden on hospitals. This study evaluates the cost consequences of this Centre by comparing healthcare service use under this new model of care compared to service use under standard clinical practice after one year of operation to determine savings to the health system.

**Methods:**

Admitted patient, non-admitted and emergency department patient records relating to chronic wounds between 2018 and 2024 were analysed to determine service use costs, number of chronic wound admissions, length of stay, non-admitted services and emergency department presentations. Regression was used to control for patient mix, and records from a neighbouring LHD utilising the standard clinical care model was used as a control for this study.

**Results:**

We estimated that with the Wound Care Command Centre™, in 2023 there were up to 97 chronic wound admissions prevented, 943 hospital days averted due to earlier discharges, 308 more non-admitted service events and 208 more emergency department presentations in Sydney LHD, compared to expected levels under standard clinical practice models. This was consistent with reduced prevalence of complex cellulitis admissions in Sydney LHD and partial shifting of care from admitted to outpatient settings. Reduced hospital admissions and earlier discharges were estimated to total between $3.2 M to $4.8 M and costs of non-admitted and emergency department services were estimated to total $264k. After accounting for $1.3 M operational costs for the Command Centre over 2023, net savings were between $1.7 M to $3.3 M.

**Conclusions:**

The Wound Care Command Centre™ reduced hospital admissions by 97 individuals and shortened hospital length of stays by 1.1 day, resulting in savings up to $3.3 M for Sydney LHD. Additional benefits for patients included increased access to specialist advice through the Wound Care Command Centre™ and reduced face-to-face contact due to use of a digital platform minimising unnecessary hospital visits for patients.

**Supplementary Information:**

The online version contains supplementary material available at 10.1186/s12913-025-12969-2.

## Background

The treatment and management of chronic wounds represent a significant challenge for healthcare systems worldwide, impacting not only patient outcomes but also healthcare costs. Chronic wounds, characterised by prolonged healing times and often associated with underlying conditions such as diabetes and venous insufficiency, can lead to considerable morbidity and healthcare expenditure [1–4]. Effective interventions are critical to address these issues, reduce healthcare costs, and improve the quality of life for affected individuals. Effective interventions are essential to mitigate these challenges, reduce healthcare expenditure, and enhance the quality of life for affected individuals. Managing these wounds is particularly complex in hospital settings, where patients may be vulnerable due to immobility, age, or comorbidities [5]. In community settings, limited access to specialised wound care services exacerbates disparities in treatment and outcomes [6]. Individuals living in rural or underserved areas often face barriers such as geographical distance to care facilities, a lack of local healthcare providers trained in wound management, and socioeconomic factors that hinder their ability to seek timely treatment [7, 8].

Chronic wounds affect an estimated 1.51 to 2.21 individuals per 1,000 worldwide, though prevalence rates fluctuate widely due to differences in patient demographics, healthcare practices, and resource availability. In the United States [3], estimates indicate that chronic wounds affected between 8.2 million and 10.5 million Medicare beneficiaries annually between 2014 and 2019, with costs reaching up to $29.7 billion each year [9]. In Australia, estimates suggest that chronic wounds affect over 400,000 individuals annually, costing the healthcare system upwards of AUD 3 billion each year [10]. These costs arise from direct treatment expenses, longer hospital stays, and complications associated with poorly managed wounds, including infections and subsequent surgeries [11].

Patients with chronic wounds often endure pain, decreased mobility, and a diminished quality of life [12]. These burdens can lead to increased lengths of hospital stays (LOS) and greater utilisation of healthcare resources [12]. The financial implications of chronic wound management extend beyond immediate treatment costs, as they also encompass the broader impact on healthcare delivery systems. Graves and Zheng highlighted that chronic wounds in Australia have significant implications for healthcare delivery, particularly hospital settings, and for pressure injuries [13]. Kapp and Santamaria further explored the out-of-pocket costs and quality-of-life impacts for individuals with chronic wounds, finding that patients spent an average of AUD$2475 on dressings and experienced a reduced quality-of-life, especially younger patients [14, 15]. Queen and Harding’s analysis of the possible costs of chronic wound care and clinical outcomes globally identified that hospitalisation rates for chronic wounds in Australia were low, but once admitted, patients faced prolonged stays and a significant risk of amputations, particularly for diabetic foot ulcers [16]. In fact, Australia is one of the top ten nations in terms of provision of wound care [16], with $5.1404 billion spent on wound care in 2019 [17]. These studies collectively emphasise both the economic and personal toll of chronic wounds. In light of these challenges, it is clear that current models to deliver wound care are both costly to the health system and impact patient outcomes negatively justifying a need for more innovative care models to enhance clinical outcomes while managing wounds cost-effectively.

In 2023, a local health district in New South Wales implemented a novel model of care for chronic wound management, integrating a digital wound care platform with a centralised wound specialist centre - the Wound Care Command Centre™ [18, 19]. This initiative sought to address the disparity in access to care for individuals with chronic wounds and minimise the need for patients to access care for chronic wounds in the acute setting while streamlining assessment and treatment processes. As found by Graves and Zheng, in Australia, most of the costs of chronic wounds are incurred in the hospital system as compared to community care services [13]. It also aimed to promote real-time data sharing and collaboration among healthcare professionals, ultimately reducing healthcare costs associated with chronic wounds. The increasing focus on digital health technologies in wound care reflects their significant potential to improve on patient outcomes, particularly through advancements in wound imaging and measurement and access to care, although further research is needed to enhance clinical efficacy in their implementation [18, 20–22].

Despite the promising advantages of technological integration in wound care, a significant gap exists in understanding its economic impact compared to traditional methods [23]. A recent study by Brain et al. employed a decision-analytic model to assess the cost-effectiveness of a specialist wound care clinic versus standard practice for chronic wound management in Australia, highlighting significant cost savings and improved patient outcomes [24]. The authors emphasised the inefficiencies inherent in current care models and the need for further cost analysis. Given the increasing prevalence of chronic wounds and the associated economic burden on healthcare systems, understanding the costs and benefits of innovative care models is crucial for informing policy decisions. However, no comprehensive study has yet evaluated the costs and outcomes of a large-scale digital wound care model.

## Methods

### Aim

This study aimed to estimate the cost consequences of the Wound Care Command Centre™ by comparing healthcare service use for chronic wound patients with a new model of care implemented in Sydney LHD, compared to their expected service use under standard practice.

### Study design

This cost-consequence study evaluated changes in healthcare service use for patients with chronic wounds during the intervention period (2023), compared to their expected service use under standard practice in the same local health district (LHD 1, Sydney). Data from a neighbouring local health district (LHD 2, South Western Sydney) was used as an additional control for system-wide changes over time. We estimated the change in service use in 2023 across hospital admissions, non-admitted wound management clinics and emergency department (ED) care, using patient episodes occurring between 1 February 2018 and 29 February 2024 across the two LHDs.

### Study setting

#### Study location

Both LHDs examined in the study offer extensive adult health services, including cardiothoracic and vascular care, ensuring a comparable baseline for analysis. LHD 1 provides secondary, tertiary, and quaternary care, and five community health centres, reporting 163,464 patient admissions, 1,500,342 outpatient visits and 174,474 ED presentations in 2023. LHD 2 operates 14 community health centres focused on prevention, early intervention, and rehabilitation. LHD 2 operates six hospitals and numerous community-based services. In the 2023/2024 FY, there were 316,791 ED presentations; 392, 913 primary and community health occasions of service, with 258, 910 patients admitted across the hospitals.

#### Intervention– the Wound Care Command Centre™

The Wound Command Centre™ is a new Model of Care that offers a person-centred nurse-led wound specialist service. It offers access to multidisciplinary care and advice fostering collaboration between hospitals, specialist services, and community care.

There are four key components to the model of care:

- Prompt access to, and prompt response from, specialist wound care: patients can access expert wound care via both face-to-face and real time audio-visual communication
- Prompt advice/consultation for the clinician. Clinicians can access expert advice on wound care for their patients
- Access to Multidisciplinary care; For example, Diabetic foot ulcer, involvement of High Risk Foot clinic
- A purpose-designed digital app; eMR-integrated, HIPAA compliant with real-time wound imaging to aid decision making and secure messaging to encourage partnership in patient care for both patients and their specialists/GPs;
- Multiple connections (virtual and face-to-face) between healthcare professionals, patients and carers to enhance shared care with all parties able to monitor and treat the wound remotely;
- Data systems that intricately link wound healing data, that is provided by the digital app, with comprehensive medical records allowing for benchmarking progress, providing a standardised measure to assess treatment effectiveness and patient outcomes and to share this data among healthcare professionals.

The Centre is part of RPA Virtual Hospital, Australia’s first ever virtual hospital, which enhances access for both treating clinicians and patients through a hybrid (virtual and face-to-face) service. People with hospital admissions for chronic wounds are eligible for the program and can be referred to the service or refer themselves. The new model of care aims to support earlier discharge from hospital while ensuring prompt access to community-based care. This approach provides patients with wound management advice and treatment at home, preventing wounds from progressing and requiring intervention in acute care facilities.

There is no set timeframe regarding how often patients are reviewed as wounds are reviewed as often as necessitated per clinical judgement regarding the wound’s healing progression and the complexity of the wound. The wound may not necessarily be reviewed by the same treating clinician at each consult. Treatment options are determined by the wound type, severity, co-morbidities, availability of wound care products, as well as patient compliance/acceptability.

#### Standard practice

In LHD 1, patients with chronic and/or complex wounds discharged from hospital into the community under standard evidence-based care models, receive care through specialist community health services. In primary care facilities (general practices) across New South Wales, patients generally must either wait for an appointment with their treating practitioner for wound follow-up or, for those with mobility challenges, for the general practitioner/health professional to visit them, which has potential to result in delays to care. When discharged from acute care services, patients are typically seen within two weeks of discharge or earlier if clinically indicated, if their treating practitioner refers them to community nursing services. Care plans are developed during visits to monitor wound status, and for complex or non-healing wounds, the community nurse may request a joint visit with a wound specialist nurse. Residential aged care facilities experience varied access to wound management services, with inconsistent approaches to managing chronic wounds across these settings.

### Data preparation and patient cohort

All dollar amounts are presented in Australian Dollars. Data for this study were extracted by the Performance Units of the LHDs, who routinely collect data on wound management activities, healthcare utilisation, and costs associated with wound care across the district. The administrative data were de-identified before analysis. This data was used to estimate service utilisation in LHD 1 with the new model of care at the Wound Care Command Centre™ versus standard practice. The specific data on healthcare utilisation collected from the LHD Performance Units included:

- *Inpatients*: Hospital admissions data for patients with chronic wound diagnoses, including care type, length of stay (LOS), diagnosis codes, classification, National Weighted Activity Unit (NWAU) and readmissions.
- *Outpatients (Non-Admitted Patients)*: Service event data for patients undergoing wound management at an outpatient setting, including NWAU.
- *ED Patients*: ED presentation data, including principal diagnosis and NWAU.

Our analysis focuses on patients with hospital admissions for ongoing or recurring chronic wounds. To identify this group, we started with a broader group of patients that included any hospital admissions with an ICD-10-AM (International Statistical Classification of Diseases and Related Health Problems, Tenth Revision, Australian Modification) diagnosis code (primary or secondary) relating to a chronic wound (Additional file 1). We then subset this group to define our patient cohort as those with chronic wounds that had not healed within 30 days.

As the actual healing time for wounds is not recorded in administrative data, we used a similar methodology to the New South Wales Ministry of Health’s ‘Leading Better Value Care’ wound management initiative to identify this cohort. Specifically, we defined a ‘chronic wound series’ as a set of admissions with chronic wound diagnosis codes, where patients had one or more subsequent admissions (‘re-admissions’) within 180 days of their initial admission, or an admission with a length of stay greater than 30 days. Some patients had more than one chronic wound series if there was a break between their admitted episodes of more than 180 days.

We retained all non-admitted wound management (tier 2 clinic 40.13 [25]), service events and ED records for patients with a chronic wound series, conditional on the records falling within the total series time period. Additionally, we flagged any LHD 1 records relating to High-Risk Foot (HRF) services for diabetic patients in the admitted data, using ICD-10-AM codes relating to diabetes-related infections or ulcers of the foot or lower limb (Additional file 2). Our analysis largely excluded this group, as there is a separate program relating to HRF wounds in LHD 1 that these patients may also be eligible for.

Our study is reliant on the historical data supplied by the Performance Units of the two LHDs. This is discussed further, alongside other limitations, in section Study Limitations.

### Statistical analysis

#### Descriptive statistics

We used descriptive statistics to understand the demographic characteristics of all chronic wound admissions (those with a chronic wound diagnosis) and our subset of ongoing or recurring chronic wounds (those with a LOS over 30 or a readmission within 180 days), excluding HRF patients. This included distributions of admissions by gender, age and discharge year across the two LHDs.

#### Modelling

Regression models were used to estimate: (1) the likelihood of a chronic wound admission escalating into an ongoing or recurring chronic wound; (2) the length of stay (LOS) per admission for ongoing or recurring chronic wounds; (3) the number of non-admitted wound management service events associated with the patient cohort; and (4) the number of ED presentations associated with the patient cohort. In all models, age, sex, LHD and discharge year were controlled for via inclusion in the model as predictors. Additionally, in the admitted patients model (models 1 and 2) case mix was controlled for by accounting for HRF records and diagnosis (measured using Adjacent Australian Refined Diagnosis Related Groups, AR-DRG). The number of admitted episodes in the associated chronic wound series were accounted for in models (3) and (4).

Each of the four models included an interaction term between the 2023 year and LHD 1, to allow for changes in health service use under the new model of care in 2023 compared to standard practice. The models were used to estimate service use in LHD 1 over 2023 for actual patients on two bases: firstly, based on historical service use up to the end of 2022, and secondly, allowing for a change in service use in 2023. The difference between the two bases provides an estimate of the change in 2023 (with the new model of care implemented), after accounting for factors such as patient age and diagnosis.

Additionally, changes in the number of non-admitted service events and ED presentations not associated with ongoing or recurring chronic wound patients were investigated, to determine whether there were any related trends in these areas.

A key limitation of the analysis is this is an aggregate approach to measuring changes in service use relating to chronic wound series in LHD 1 in 2023. We have attempted to control for changes in patient mix and system-wide changes, however there may be other changes coincident with the new model of care that resulted in changes to health service use in LHD 1 over 2023. Additionally, our analysis was restricted to service use related to the provided set of ICD-10 codes. Any potential impacts on service use outside of these codes were not analysed.

#### Costs and savings

The gross savings or costs associated with each component were calculated based on the change in: (1) hospital days due to the change in the number of chronic wound admissions; (2) hospital days due to the change in the average length of stay per chronic wound admission; (3) non-admitted wound management service events; and (4) ED presentations. Each component was multiplied by the average NWAU (per day for admissions; per event for non-admitted and ED events) and the New South Wales state efficient price for 2022-23, before being added together to determine total gross savings.

For component (1), an upper bound and a lower bound were calculated based on different assumptions. The upper bound assumes that for the admissions prevented from becoming an ongoing or recurring chronic wound, the full length of stay was avoided for all admissions (i.e. all episodes prevented were readmissions). The lower bound assumes that for the admissions prevented from becoming an ongoing or recurring chronic wound, that the full length of stay was only avoided for the proportion assumed to be readmissions, based on the number of chronic admissions per chronic wound series (i.e. the proportion assumed to be initial admissions were shifted to being non-recurring chronic wound admissions, rather than being fully prevented).

The program expenditure for 2023 was calculated as the sum of all operational costs for the new model of care and Wound Care Command Centre™. This included 12 months of costs for mobile devices, software licences and staff. This figure was subtracted from the total gross savings to give the net savings of the program in 2023. Establishment costs for 6 months prior to the program were also calculated, however these were noted as a separate figure, as they should be distributed over the lifetime of the centre rather than attributed to a single year.

### Ethics

This project has been approved by the *Sydney Local Health District Human Research Ethics Committee (RPAH Zone) (2019/ETH12459*,* protocol number X19-0307).* The study was part of a larger research initiative consisting of three stages. This cost study represents the third stage. Data from patients in stages 1 and 2 were utilised, as participants had already provided informed consent for their medical records to be accessed and their information to be used for research purposes.

Additional aggregated data were collected from the Performance Unit at a health system level, ensuring that patients were unidentifiable. A waiver of consent was approved for the data, as the Ethics Committee deemed this component to pose no more than low risk, as it involved a retrospective audit of medical records and healthcare utilisation data at the health system level. The study was conducted in accordance with the National Health and Medical Research Council’s National Statement on Ethical Conduct in Human Research [26].

## Results

We have estimated the change in service use in LHD 1 over 2023, compared to what we would expect based on historic trends. Records from LHD 2 provide a control for system-wide changes over 2023.

### Descriptive statistics

Table 1 shows the distribution of admitted care episodes (excluding HRF episodes) in LHD 1 and LHD 2 by demographic factor, for all admissions with a chronic wound diagnosis and for those that are ongoing or recurring. The distributions are generally similar, although LHD 1 sees a slightly different age profile, with more ongoing or recurring chronic wounds for patients aged < 80 years, compared to LHD 2. There are also fewer records in 2018 for LHD 2, which we suspect is due to partially incomplete data. Overall, ongoing or recurring chronic wound admissions account for a similar proportion of all chronic wound-related admissions across both LHDs, with 23% in LHD 1 and 28% in LHD 2.

**Table 1:** Number of admitted episodes by chronic wound cohort, LHD and demographic factors

Demographic factor	All admissions with chronic wound diagnosis (excluding High Risk Foot [HRF] episodes)		Ongoing or recurring chronic wounds* (excluding HRF episodes)	
	LHD 1	LHD 2	LHD 1	LHD 2
Gender:				
Female	11,813 (46%)	17,248 (44%)	2,767 (48%)	4,730 (43%)
Male	13,817 (54%)	21,715 (56%)	3,006 (52%)	6,278 (57%)
Age group:				
< 50 years	9,192 (36%)	12,606 (32%)	1,107 (19%)	1,992 (18%)
50–59 years	3,042 (12%)	4,833 (12%)	644 (11%)	1,395 (13%)
60–69 years	3,257 (13%)	6,159 (16%)	877 (15%)	2,200 (20%)
70–79 years	3,854 (15%)	7,025 (18%)	1,158 (20%)	2,652 (24%)
80 + years	6,285 (25%)	8,340 (21%)	1,987 (34%)	2,769 (25%)
Discharge year:				
2018	4,479 (17%)	3,704 (10%)	946 (16%)	1,001 (9%)
2019	4,770 (19%)	7,618 (20%)	1,048 (18%)	2,186 (20%)
2020	4,210 (16%)	7,108 (18%)	973 (17%)	2,065 (19%)
2021	3,963 (15%)	6,624 (17%)	1,004 (17%)	1,896 (17%)
2022	3,881 (15%)	6,661 (17%)	942 (16%)	1,890 (17%)
2023	4,327 (17%)	7,248 (19%)	860 (15%)	1,970 (18%)
Total	25,630	38,963	5,773	11,008

* Chronic wound admissions with a length of stay > 30 days or a readmission within 180 days

Table 2 shows the demographics of the Wound Care Command Centre™ patients admitted in 2023.

**Table 2:** Demographics of Wound Care Command Centre^™^ patients (*n* = 149) in 2023

Variable	Number of patients (%)
Gender	
Female	81 (54.4)
Male	68 (45.6)
Age group	
< 50 years	19 (12.8)
50–59 years	12 (8.1)
60–69 years	54 (36.2)
70–79 years	26 (17.4)
ICD-10 Primary Wound Type	
L89 – Pressure Injury	36 (24.2)
I83/I86/I87 – Varicose veins of lower extremities/Varicose veins of other sites/Other disorders of veins	26 (17.4)
T81 - Complications of procedures, not elsewhere classified	24 (16.1)
E09/E10/E11/E13/E14 - Diabetes Mellitus	14 (9.4)
T14 - Injury of unspecified body region	9 (6.0)
R23.4 - Changes in skin texture	8 (5.4)
L98 - Other disorders of skin and subcutaneous tissue, not elsewhere classified	5 (3.4)
L97 - Ulcer of lower limb, not elsewhere classified	5 (3.4)
L02 – Abscess	4 (2.7)
L24 - Irritant contact dermatitis	4 (2.7)
T89 - Open wound with complication of foreign body, infection and delayed healing/treatment	4 (2.7)
M79 - Other soft tissue disorders, not elsewhere classified	3 (2.0)
T22 - Burn of shoulder and upper limb, except wrist and hand	1 (0.7)
L08 - Other local infections of skin and subcutaneous tissue	1(0.7)
A18.4 - Tuberculosis of skin and subcutaneous tissue	1(0.7)
T24 - Burn of hip and lower limb, except ankle and foot	1(0.7)
B87 - Myiasis	1(0.7)
L03 – Cellulitis	1 (0.7)
S80.82 - Blister of lower leg	1 (0.7)

### Modelling

Table 3 summarises the estimated differences in key figures across the admitted, non-admitted and ED patient records, under the new model of care compared to standard practice. Under the new model of care, we have estimated a reduced number and length of admissions for patients with ongoing or recurrent current wound series, as well as an increased number of non-admitted and ED events associated with these patients. Further details on how these figures were estimated are described below.

**Table 3:** Actual figures and estimated changes in health service use for ongoing and recurring chronic wound patients in LHD 1 in 2023

Stream	Figure	Actual	Estimate under standard practice	Estimate under new model of care	Difference in estimates
AP	Number of admissions in a chronic wound series	860	951	854	-97
	Number of initial admissions*	409	452	406	-46
	Number of readmissions*	451	499	448	-51
	Average length of stay per admission (days)	24.4	25.6	24.5	-1.1
NA	Number of NA wound management service events associated with patient cohort	1,188	880	1,188	308
ED	Number of ED presentations associated with patient cohort	863	655	863	208

*AP* Admitted patients, *ED* Emergency department, *NA* Non-admitted patients

* Calculated based on the observed number of chronic admissions per chronic wound series of 2.10 (1.1 readmissions for every admission)

**Fig. 1:**
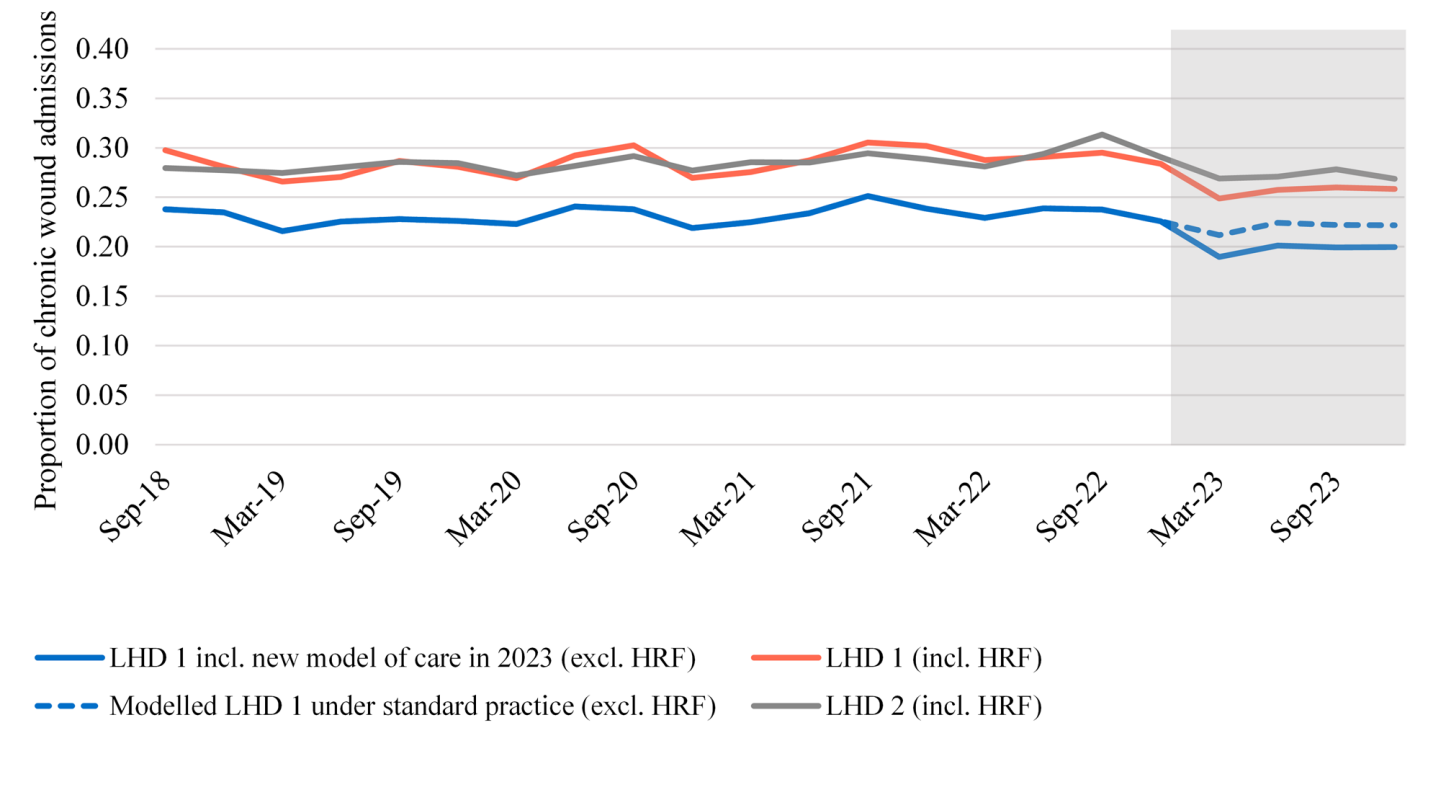
Proportion of chronic wound admissions that become an ongoing or recurring chronic wound* *Chronic wound admissions with a length of stay > 30 days or a readmission within 180 days

Figure 1 shows the proportion of admissions with a chronic wound diagnosis which become an ongoing or recurring chronic wound. This is shown for LHD 1 and LHD 2 over time, including HRF records. The two LHDs generally follow a similar trend, although the rate has decreased in LHD 1 over 2023 more than in LHD 2.

Figure 1 also shows the proportion of chronic wound admissions that are part of an ongoing or recurring chronic wound series for LHD 1 (excluding HRF records) under the new model of care compared to expected rates based on information to the end of 2022 (modelled LHD 1 under standard practice, excl. HRF). Under standard practice, we would have expected the rate over 2023 to be around 0.22. With the new model of care, the rate is closer to 0.20. This equates to an estimated 97 admissions being prevented from becoming part of an ongoing or recurring chronic wound series over 2023, with the new model of care implemented. With an average length of stay (ALOS) of 25.6 days, this equates to at least 1,352 fewer admitted days (assuming a proportion of the prevented episodes were initial admissions) and up to 2,480 fewer admitted days (assuming all prevented episodes were readmissions) as a result of avoided chronic wound admissions.

**Fig. 2:**
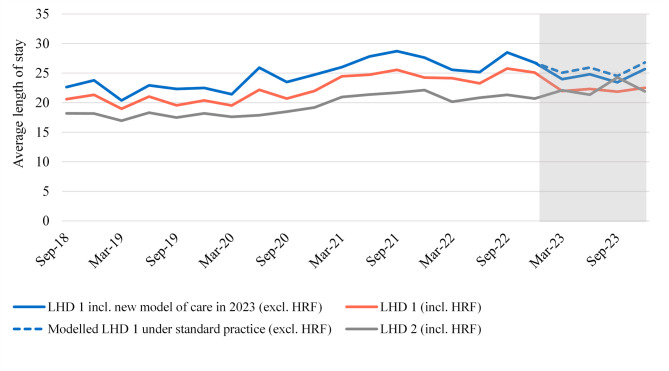
Average length of stay per admission in an ongoing or recurring chronic wound series

Figure 2 shows a similar comparison for the average length of stay per admission in an ongoing or recurring chronic wound series.

Comparing LHD 1 and LHD 2, the ALOS is generally higher for LHD 1. We explored this and found it was likely due to differences in case-mix and coding practices between the two LHDs. The difference between these LHDs is particularly driven by the higher number of episodes in LHD 1 with AR-DRGs G01A (rectal resection), U61A (schizophrenia disorders) and Z64A (other factors influencing health status), all of which have relatively higher ALOS.

Comparing the predicted ALOS for LHD 1 (excluding the HRF cohort) under the new model of care and under standard practice, we estimated 1.1 days per admission were avoided over 2023. Multiplying this difference by the 854 remaining chronic wound admissions, this equates to 943 fewer admitted days.

**Fig. 3:**
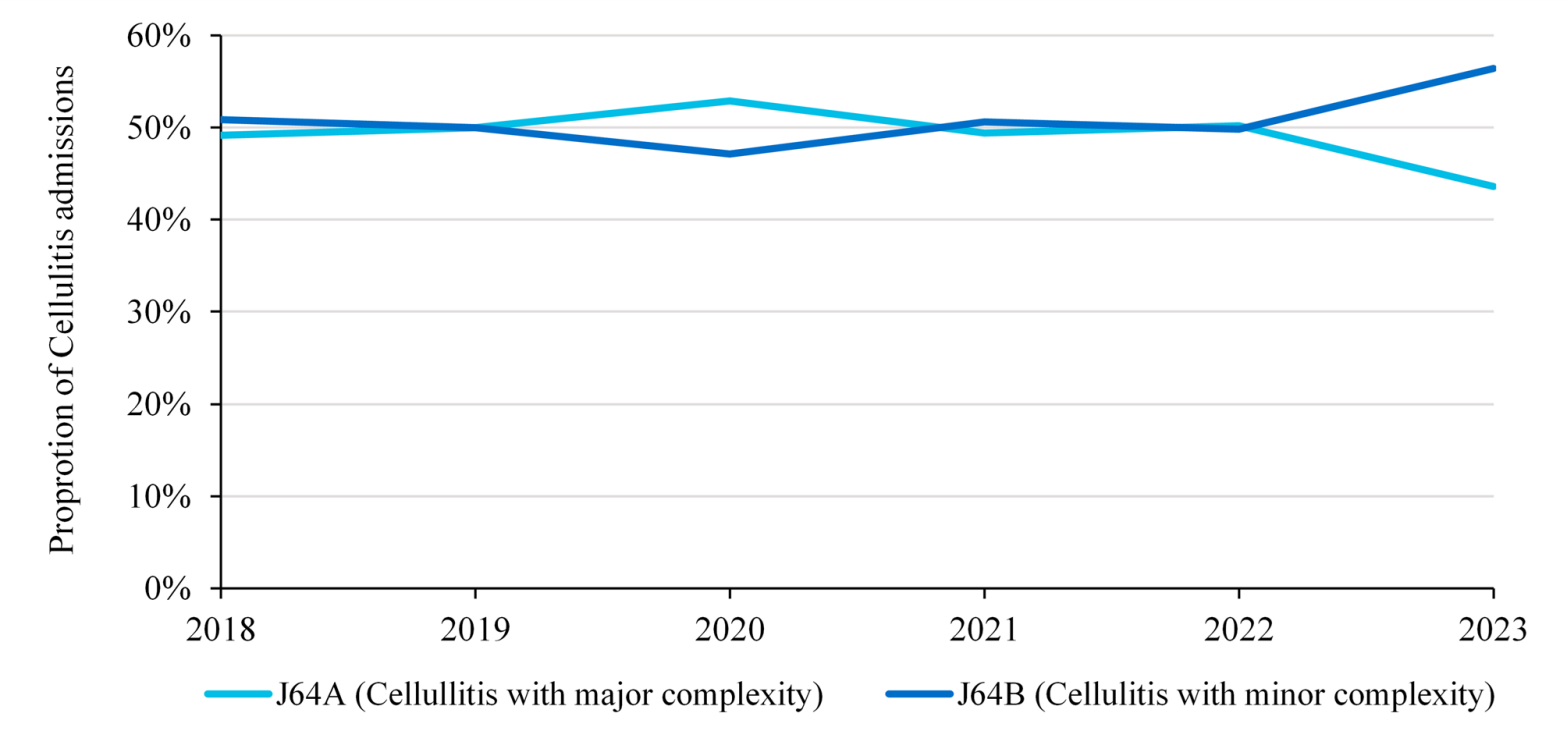
Proportion of ongoing or recurring chronic wound admissions by complexity, for adjacent AR-DRG J64 (cellulitis)

We looked into potential causes of the decrease in ALOS per chronic wound admission and found that on average, admissions in the period with the new model of care were less complex. This was largely driven by a shift towards lower complexity AR-DRGs amongst patients with cellulitis, who make up approximately 30% of chronic wound admissions in LHD 1. This is evident in Fig. 3, which shows the distribution of ongoing or recurring chronic wound admissions in LHD 1 by AR-DRG over time, for Adjacent AR-DRG J64.

**Fig. 4:**
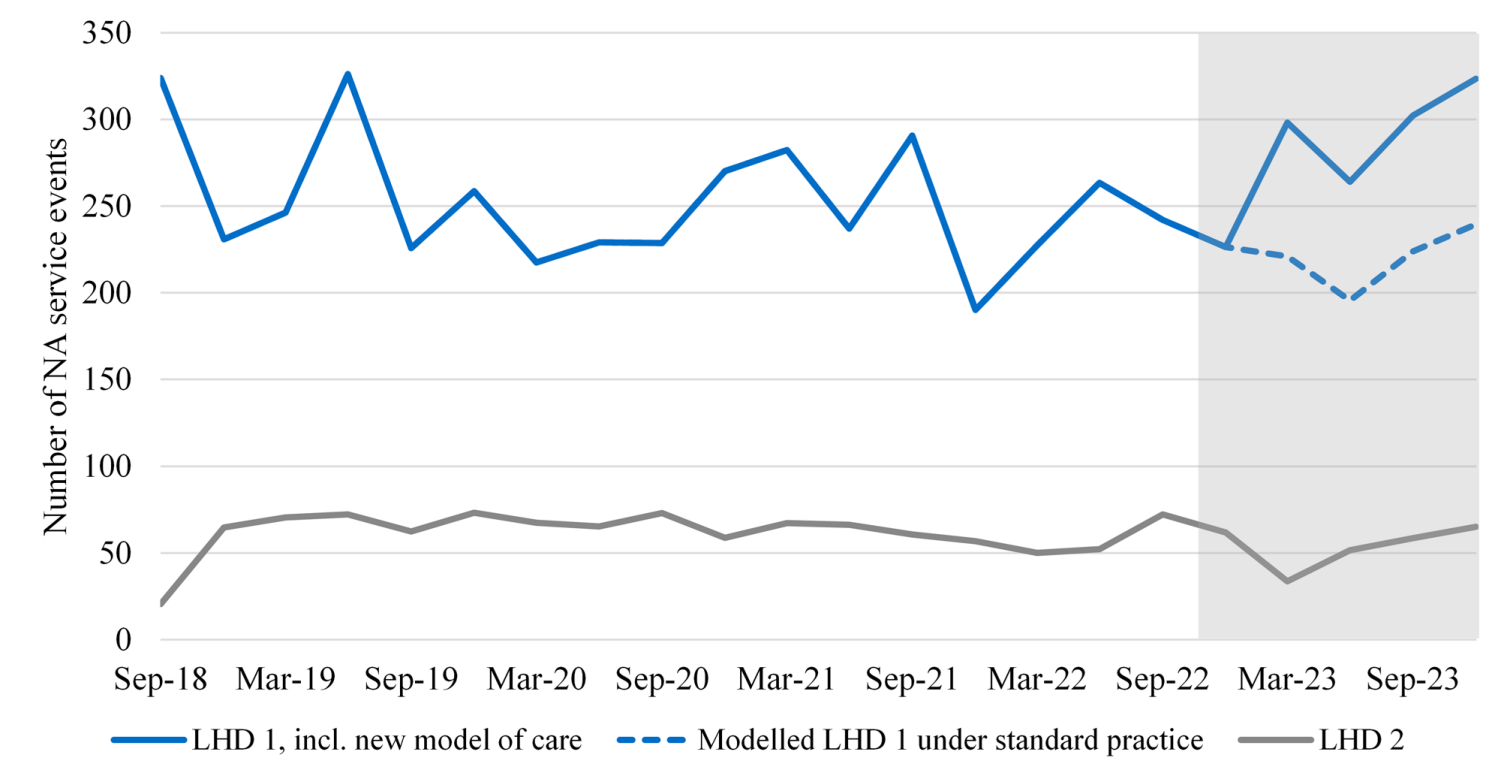
Non-admitted wound management service events for patients with an ongoing or recurring chronic wound series

Figure 4 shows a similar comparison to Figs. 1 and 2 but for the number of non-admitted service events at wound management clinics (tier 2 clinic 40.13 [25]), for patients with an ongoing or recurring chronic wound series.

The average number of service events over time is very different for LHD 1 and LHD 2, making it difficult to form exact estimates. However, when comparing the model predictions under the new model of care and standard practice, there is a definite increase in the number of non-admitted service events at wound management clinics in LHD 1 over 2023. This is consistent with some activity from chronic wound series patients being shifted from admitted settings to outpatient settings. We estimate 308 additional service events occurred over 2023 with the new model of care operating, although there is some uncertainty in this figure due to the large differences between LHD 1 and LHD 2.

**Fig. 5:**
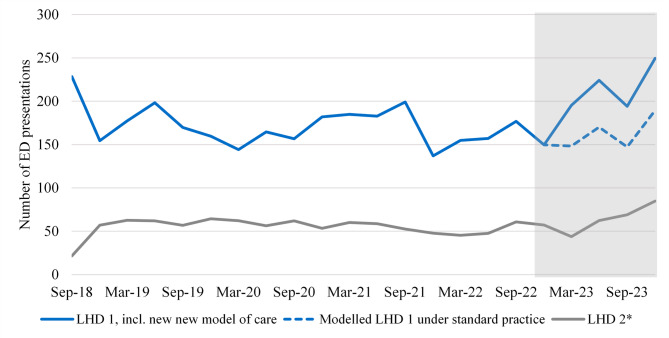
Number of ED presentations for patients with an ongoing or recurring chronic wound series *Includes three out of six emergency departments

Figure 5 shows similar information for ED presentations.

The average number of presentations over time is very different for LHD 1 compared to LHD 2, however the LHD 2 data is restricted to three of the six EDs in the LHD. Similarly to non-admitted activity, Fig. 5 shows an increase in ED presentations with the new model of care operating over 2023 compared to standard practice. We estimate an additional 208 ED presentations occurred over 2023 with the new model of care compared to standard practice. As with outpatient activity, this is consistent with a partial shifting of care from admitted settings to other healthcare settings.

### Costs and savings

Table 4 shows how the estimations derived from the regression models translate into service use costs and savings. Overall, we can see that the benefit of the reduced admitted days vastly outweighing the increased outpatient and ED activity.

**Table 4:** Gross program savings, by savings component

Stream	Savings component	Calculation	Savings
AP	Savings associated with the change in the number of chronic wound admissions	Lower bound: 51 re-admissions^(a)^ prevented x 0.2768 NWAU per day^(b)^ x ALOS 25.6 days x $5,095 + 46 initial admissions^(a)^ x 0.2768 NWAU per day^(b)^ x 1.1 day per admission saved x $5,095 Upper bound: 97 admissions prevented x 0.2775 NWAU per day^(b)^ x ALOS 25.6 days x $5,095	$1,906,318 to $3,497,861
	Savings associated with the change in the ALOS per chronic wound admission	854 chronic wound admissions x 1.1 days saved per admission (943 admitted days saved) x 0.2775 NWAU per day^(b)^ x $5,095	$1,330,017
NA	Savings associated with the change in the number of NA wound management service events associated with admitted chronic wound patients	308 additional NA service events x 0.0383 NWAU^(c)^ x $5,095	-$60,133
ED	Savings associated with the change in the number of ED presentations associated with admitted chronic wound patients	208 additional ED presentations x 0.1922 NWAU^(d)^ x $5,095	-$203,550
Total gross savings			$2,972,642 to $4,564,195

*ALOS* Average length of stay, *AP* Admitted patients, *ED* Emergency department, *NA* Non-admitted patients, *NWAU* National Weighted Activity Unit

^(a)^ Calculated based on the observed number of chronic admissions per chronic wound series of 2.10 (1.1 readmissions for every admission)

^(b)^ Calculated based on the observed total admitted NWAU divided by the total bed days for the patient cohort over 2023

^(c)^ The 2022-23 price weight for tier 2 clinic 40.13

^(d)^ Calculated based on the observed average ED NWAU for the patient cohort over 2023

Table 5 outlines the 2023 operational costs associated with the intervention program, by expenditure category– these total approximately $1.3 M. When subtracted from the gross savings figures, this results in net savings of at least $1.7 M and up to $3.3 M.

**Table 5:** 2023 Operational costs, by expenditure category (calendar year)

Expenditure category	Item	2023 costs
Software and hardware costs	Mobile devices	$33,936
	Software license* (1st year)	$169,905
Operational staff costs	Program manager	$164,643
	Clinical nurse consultant	$134,356
	Nurse practitioner	$149,038
	Senior project officer (part time)	$44,535
	Product analyst	$101,834
	Digital health coach	$77,175
	Senior computer operator	$31,748
Management staff costs	Project manager	$146,672
	Project officer	$164,185
	Additional ICT support	$62,817
Total		$1,280,842

*ICT* Information and Communication Technology

^*^ We note that the distribution of licensing cost between 2023 costs and start-up costs is an estimate

In addition to these operational costs, the initial investment costs to set up the program, outlined in Table 6, totalled approximately $980k. These costs were not included in the net savings calculations, as they are intended to be distributed over the lifetime of the program. However, even if the full amount of the establishment costs was accounted for in 2023, the program would still have a net benefit between $0.7 M and $2.3 M.

**Table 6:** Initial investment costs, by expenditure category

Expenditure category	Item	Setup costs
Facility costs	Dedicated facilities (4 pods)	$26,680
Promotional costs	Promotional video	$7,160
Software and hardware costs	Computer package for telehealth	$17,795
	Software implementation	$280,000*
	Software testing	$33,541
	Mobile devices	$149,448
	Software licence (6 months)	$84,953
Operational staff costs	Program manager	$76,772
	Clinical nurse consultant	$57,581
	Nurse practitioner	$72,164
	Senior project officer	$19,902
	Product analyst	$29,134
	Digital health coach	$13,306
	Senior computer operator	$10,444
Management staff costs	Project manager	$37,941
	Additional ICT support	$62,817
Total		$979,636

*ICT* Information and Communication Technology

^*^These costs are close estimates

## Discussion

Our study aimed to explore the impact of the Wound Care Command Centre™ on service outcomes, particularly focusing on reducing hospital admissions and length of stay for chronic wound patients. We found that the new care model significantly reduced hospital admissions and length of stay, with notable demographic trends emerging across both health districts. Additionally, our analysis revealed shifts in care settings and substantial cost savings, highlighting the effectiveness of this model in improving patient outcomes and optimising healthcare resource utilisation.

### Patient cohort

The demographic trends observed in our study align with existing literature which highlights the disproportionate burden of chronic wounds, particularly among patients under 50 years and those aged 80 and older [9, 27]. In our data, patients under 50 years accounted for 36% of chronic wound admissions in LHD 1 and 32% in LHD 2, while those aged 80 and older represent a significant portion of the chronic wound population, highlighting the varied demographic factors that contribute to chronic wound prevalence.

Our study found that in both health districts, males are more likely to be admitted with a chronic wound compared with females. Research suggests this may be due to delayed healthcare-seeking behavior and higher rates of comorbidities, such as diabetes and vascular diseases, which are known risk factors for chronic wounds males [28]. A study by Carter and colleagues, which analyzed Medicare claims data from 2014 to 2019 found that 16.3% of Medicare beneficiaries had chronic wounds, with a notably higher prevalence observed among males under the age of 65 compared to females [9]. On the other hand, the Wound Care Command Centre^™^ had a higher prevalence of female patients with chronic wounds compared to males. Interestingly, our data also shows that younger patients (under 50 years) represent a significant portion of chronic wound admissions, with 36% in LHD 1 and 32% in LHD 2. This demographic may reflect a different set of risk factors, such as trauma, surgical wounds, or underlying health conditions, that contribute to chronic wound development in a younger population. The high prevalence among individuals aged 80 and older is also consistent with previous findings, emphasising the impact of aging on wound healing capacity, reduced mobility, and increased dependency on care [29].

The higher proportion of older patients in LHD 1 (34% aged 80 and older for ongoing wounds compared to 25% in LHD 2) highlights the impact of regional demographic and healthcare access differences on chronic wound care services. Despite consistent discharge rates across both districts, the stable resource allocation may struggle to meet the demands of a growing ageing population. The Wound Care Command Centre^™^ plays a pivotal role in addressing these challenges. By facilitating prompt access to care through advanced remote monitoring, real-time data analytics, and personalized care plans, it ensures that high-risk patients receive timely interventions. This approach optimizes resource utilization and improves patient outcomes. Furthermore, the Centre’s focus on innovation and clinician education empowers healthcare providers with the skills and tools necessary to manage complex wounds effectively, thereby enhancing the overall responsiveness of chronic wound care services across diverse population groups.

#### Prevention of admitted care

The results of this study suggest the new model of care implemented in LHD 1 is successful in reducing the number of admissions and length of admissions associated with chronic wounds. Combining the estimates from the regression models and subtracting the 2023 operational costs results in an overall reduction in service use of at least $1.7 M and up to $3.3 M. These reductions correspond to at least a 4.8% and up to a 9.4% reduction in service use from our baseline scenario assuming no change to the model of care. This suggests that there are large potential benefits associated with reducing the amount of unnecessary time chronic wound patients spend in hospital.

We identified two main drivers behind this reduction: firstly, the ability for patients to manage and monitor their wounds in their home environments or outpatient settings, whilst receiving specialist advice; and secondly, the shifting of cellulitis (infection of a wound) admissions in LHD 1 from major to minor complexity. The reduction in the proportion of cellulitis admissions with major complexity suggests that the new model of care may be resulting in cellulitis patients being admitted earlier (before they become complex), or some cellulitis infections being prevented altogether.

### Shifting of care

There is evidence that some of the avoided admitted patient care shifted to outpatient and emergency settings. However, the shifting of care to these settings typically reduces costs, due to differences in the NWAU between care types. The NWAU is a measure of health service activity, expressed as a common unit, which allows us to compare the clinical complexity of health services across different settings. The average hospital service is worth one NWAU, with more intensive and expensive activities worth multiple NWAU, and less intensive and expensive activities worth fractions of an NWAU.

In the case of patients with ongoing or recurring chronic wounds analysed in this study, the average NWAU was 0.2768 per admitted day, or 6.7771 per hospital admission. In comparison, the average NWAU per non-admitted service event was 0.0383 and the average NWAU per ED presentation was 0.1922. Thus, a shifting of activity from admitted care to outpatient and emergency care results in a net benefit.

For the calculation of savings attributable to the change in the number of chronic wound admissions, a lower bound and an upper bound were calculated based on different assumptions, as described in the methodology. For our lower bound scenario, where 46 initial admissions are assumed to shift from being part of an ongoing or recurring chronic wound series to being singular and less complex chronic wound admissions, we have calculated that there may be up to $162k of additional costs associated with corresponding increases in the number of non-admitted and ED events. These are excluded from our gross savings due to their inherent uncertainty, but they would not have a large impact on the total figure if they were to be included.

The shift of care to the ED has been largely driven by the inability of the Wound Care Command Centre^™^ to prescribe a range of medications or conduct necessary diagnostic studies for patients with deteriorating wounds. When such issues are identified, referrals to ED are made as a preventive measure to stop further wound deterioration. However, this practice has prompted a reassessment of the care model, with efforts now focused on reducing the frequency of ED referrals. This has been achieved by formal linkages with general practitioners from LHD 1, allowing for earlier intervention and reducing unnecessary ED visits. Furthermore, our study indicates that some patient care that previously required admission has shifted to outpatient and emergency settings, which is more cost-effective due to differences in NWAU values between care types. The ongoing analysis of these results is being used to refine care pathways, ensuring that patients are managed more effectively in the appropriate settings and reducing the need for ED referrals in the future. This shift not only improves patient access to timely care but also offers substantial financial savings, demonstrating the potential benefits of this evolving model.

### Study limitations

#### Supplied data

Our analysis has relied on historical data supplied by the Performance Units of the two LHDs. The results of the analysis rely on the completeness and accuracy of the data provided. We have not audited or verified the accuracy of the data; however, we did carry out high-level checks on the data, including checks for internal consistency, and did not find evidence of material inaccuracies. We note that the ED data for LHD 1 contained around 0.3% fewer records than aggregated reporting figures. Due to the way the data was extracted, it is possible that the missing rate is higher for the chronic wound series cohort. Our model results are based on the population of chronic wound series for which we are confident the ED data is complete. We checked the results did not materially change if we included all records available. We also note that the estimated increase in ED service use costs is small relative to the avoided admitted patient costs settings. We do not expect the small amount of potentially missing data would materially impact the conclusions.

#### Modelling approach and assumptions

The analysis takes an aggregate approach to measuring changes in service use relating to chronic wound series in LHD 1 in 2023. We have used regression to control for changes in patient mix, and we have included data from LHD 2 to provide a control for state-wide changes. We have also excluded HRF records, as these patients may be eligible for other services that also provide benefits. However, there may be other changes coincident with the new model of care that resulted in changes to health service use in LHD 1 over 2023, which have not been separately identified.

We did not separate out the patients who attended the Wound Care Command Centre™ into a separate cohort within our analysis, as we did not have access to clear program entry, re-entry or exit dates.

Our analysis was restricted to service use related to the provided set of ICD-10 codes. Any potential impacts on service use outside of these codes were not analysed. Additionally, we understand that the ICD-10-AM code version changed from Eleventh edition to the Twelfth edition from 1 July 2022, shortly before the new model of care was implemented. While we do not believe the changes impacted chronic wound codes, we have not undertaken a comprehensive assessment of the coding changes. The impact of any unexpected changes on our results would likely be mitigated by the fact that LHD 1 and LHD 2 would be similarly impacted, and therefore would control for the system-wide change.

The study cohort focuses specifically on chronic wound series (ongoing or recurring chronic wounds lasting over 30 days). This is consistent with the new model of care which focuses on supporting people with recurrent issues. We also explored whether any costs or savings could be observed relating to changes in service use for broader chronic wound admissions (not ongoing or recurring) over 2023, but did not observe any significant changes in our preliminary analysis. This supported our approach of focusing on the service use related to ongoing or recurring chronic wounds.

Finally, we were unable to control for case mix of the non-admitted patients because there was no measure of diagnosis or complexity for these patients. Further, the evaluation focused only on the first year of the model of care as the authors wanted to gain early insights before conducting a longitudinal evaluation.

## Conclusions

The Wound Care Command Centre™ in LHD 1 generated cost savings of $1.7 M to $3.3 M through reduced hospital admissions and shorter stays. These initial findings suggest that the Centre enhanced patient access to specialist care and facilitated remote monitoring, improving patient convenience and reducing reliance on hospitals. Furthermore, the Centre is linked to significant benefits in healthcare resource management and patient outcomes. These findings reflect positively on the initial year of operations of the Centre, however, a longer evaluation is required to explore whether these savings can be sustained in the long term and whether this model of care can be implemented at other sites and at scale. This research contributes to the ongoing discourse on effective chronic wound management strategies and models of care that reduce economic burden on the health system.

## Supplementary Information

Supplementary Material 1.

Supplementary Material 2.

## Data Availability

The analysis made use of LHD Performance Unit record de-identified administrative data. The datasets generated and/or analysed during the current study are not publicly available due to the associated privacy risks. Our Ethical approval specifies the governance arrangements for this data which includes access restrictions.

## Abbreviations

ALOS: Average length of stay
AR-DRG: Australian Refined Diagnosis Related Groups
AUD: Australian Dollars
ED: Emergency Department
eMR: Electronic Medical Record
GP: General practitioner
HIPAA: Health Insurance Portability and Accountability Act
HRF: High-Risk Foot
ICD-10-AM: International Statistical Classification of Diseases and Related Health Problems, Tenth Revision, Australian Modification
ICT: Information and Communication Technology
LOS: Length of stay
LHD: Local Health District
NA: Non-admitted
NWAU: National Weighted Activity Unit
