# Correction: Digitising wound care: a cost-consequence analysis of the Wound Care Command Centre™ in Australia

**DOI:** 10.1186/s12913-025-13510-1

**Published:** 2025-09-22

**Authors:** Michelle Barakat-Johnson, Meg Newton, Crispin Cayley, Michelle Lai, Laura Dixie, Nathaniel Alexander, Marc Pelusi, Jimmy Chan, Anna Cohen

**Affiliations:** 1 Nursing and Midwifery Executive Services, Sydney Local Health District, Level 11 King George V Building, Nursing Executive, Missenden Road Camperdown, Sydney, NSW Australia; 2 Susan Wakil School of Nursing and Midwifery, Faculty of Medicine and Health, University of Sydney, Camperdown, Sydney, NSW Australia; 3 School of Health and Human Sciences, University of Huddersfield, Huddersfield, UK; 4 Taylor Fry, Sydney, Australia; 5 South Western Sydney Local Health District, Liverpool Hospital Eastern Campus, Liverpool, Sydney, NSW Australia; 6 Single Digital Patient Record Implementation Authority, Chatswood, Sydney, NSW Australia; 7 RPA Virtual Hospital, Sydney Local Health District, Camperdown, Sydney, NSW Australia

**Correction to: BMC Health Services Research (2025) 25:873**

10.1186/s12913-025-12969-2

In this article, the authors reported Errors in the Abstract and in the footnote of Table 5.

The final sentence in the ‘Conclusions’ section in the Abstract was incorrectly given as:

Additional benefits for patients included increased access to specialist advice through **the** and reduced face-to-face contact due to use of a digital **platforms** minimising unnecessary hospital visits for patients.

But should have been:

Additional benefits for patients included increased access to specialist advice through **the Wound Care Command Centre™** and reduced face-to-face contact due to use of a digital **platform** minimising unnecessary hospital visits for patients.

The footnote of Table 5 was incorrectly given as:

*This cost is reflective of up to 300 users,** further costs applicable for additional users.** We note that the distribution of licensing cost between 2023 costs and start-up costs is an estimate.

But should have been:

*We note that the distribution of licensing cost between 2023 costs and start-up costs is an estimate.

The original article has been corrected.

