# Supplementary material for: Digitising wound care: a cost-consequence analysis of the Wound Care Command Centre™ in Australia
Source: BMC Health Serv Res. 2025 Jul 1;25:873. doi: 10.1186/s12913-025-12969-2 (PMC12220165; doi:10.1186/s12913-025-12969-2)
Supplement: Supplementary file 1 — Supplementary Material 1. [file 12913_2025_12969_MOESM1_ESM.docx]

**Additional File 1.** ICD-10-AM codes used to identify chronic wounds

| **Group name** | **ICD-10-AM wound codes** |
| --- | --- |
| Diabetes | E09.52, E10.52, E10.62, E10.73, E11.52, E11.62, E11.73, E13.52, E13.62, E13.73, |
| Venous | E14.52, E14.62, E14.73 |
| Cutaneous abscess | I83.0, I83.2, I86.8, I87.0, I87.2 |
| Cellulitis | L02.0, L02.1, L02.2C, L02.3, L02.40, L02.41, L02.42, L02.43, L02.8, L02.9 |
| Infection | L03.01, L03.02, L03.12, L03.13, L03.14, L03.19, L03.2, L03.3, L03.8, L03.9 |
| Radiation ulcer | L08.0, L08.1, L08.8, L08.9 |
| Gangrene | L59.8 |
| Pressure injury | L88 |
| Granuloma | L89.xx (all) |
| Lupus | L92.1, L92.2, L92.3, L92.8, L92.9 |
| Vasculitis | L93.x, L94.x, L95.0 |
| Foot ulcer | L95.1, L95.8, L95.9 |
| Chronic ulcer | L97.0, L97.8, L97.9 |
| Obstetric | L98.4 |
| Gangrene | O86.0, O90.0, O90.1 |
| Skin tear | R02 |
| Procedure | R23.4 |
| Complication open | T81.3, T81.4 |
