# Supplementary material for: Digitising wound care: a cost-consequence analysis of the Wound Care Command Centre™ in Australia
Source: BMC Health Serv Res. 2025 Jul 1;25:873. doi: 10.1186/s12913-025-12969-2 (PMC12220165; doi:10.1186/s12913-025-12969-2)
Supplement: Supplementary file 2 — Supplementary Material 2. [file 12913_2025_12969_MOESM2_ESM.docx]

**Additional File 2.** ICD-10-AM codes used to identify HRF-related wounds

| **Group name** | **Sub-group** | **ICD-10-AM HRFS wound codes** |
| --- | --- | --- |
| Diabetic patients | Diabetes | Any of E10.x, E11.x, E13.x or E14.x |
| Foot-related infections or ulcers | Infection and/or ulcer | E10.73, E11.73, E13.73, E14.73, L03.02, L03.11, L03.13, L03.14, L97.x |
|  | Peripheral vascular disease | E10.51, E10.52, E11.51, E11.52, E13.51, E13.52, E14.51, E14.52 |
|  | Peripheral neuropathy | E10.42, E11.42, E13.42, E14.42, E10.43, E11.43, E13.43, E14.43, E10.61, E11.61, E13.61, E14.61, E10.71, E11.71, E12.71, E13.71, E14.71 |

HRF = High-Risk Foot
